# Supplementary material for: FixNCut: single-cell genomics through reversible tissue fixation and dissociation
Source: Genome Biol. 2024 Mar 29;25:81. doi: 10.1186/s13059-024-03219-5 (PMC10979608; doi:10.1186/s13059-024-03219-5)
Supplement: Supplementary file 1 — Additional file 1: Supplementary Figures. Supplementary Figures 1-6 and Supplementary figure legends. [file 13059_2024_3219_MOESM1_ESM.zip › additional files figures docx and pdf/FixNCut_AdditionalFile1_text.docx]

***Additional File 1: Supplementary Figures***

**Figure S1.** **FixNCut protocol tested in human PBMCs.** (**a**) Bright-field microscopy images of fresh (**left**) and fixed (**right**) human PBMCs. (**b**) cDNA profiles of human PBMCs in fresh (**top**) and fixed (**bottom**) samples. (**c**) Final GEX library BioA profiles of human PBMCs in fresh (**left**) and fixed (**right**) samples. (**d**) Comparative analysis of the number of detected UMIs (**left**) and genes (**right**) for each cell barcode based on sequencing reads using a linear model. (**e**) Distribution of main QC metrics (number of UMIs, genes, and percentage of mitochondrial genes) by protocol. (**f**) Distribution of main QC metrics by cell type and protocol. (**g**) Overlap of highly variable genes (HVGs) shared between fresh and fixed human PBMCs, considering 3000 HVGs computed for each sample independently. (**h**) Linear regression model comparing the average gene expression levels of expressed genes by cell type. The coefficient of determination (R2) computed with Pearson correlation and the corresponding p-value are indicated. (**i**) Dot plot showing the average expression for top genes (*x-axis*) across all 19 cell types (*y-axis*). Dot size represents the percentage of cells in a cluster expressing each gene, and the color indicates the average expression level. (**j**) Score of sampling-time gene signature [[4]](https://www.zotero.org/google-docs/?VlCxmh) for fresh and fixed human PBMCs across cell populations. Statistical analysis between fixed and fresh cells was performed using Wilcoxon signed-rank test; significance results are indicated (ns, p>0.05, * p<=0.05, ** p<=0.01, *** p<=0.001, **** p<=0.0001). (**k**) Overlap between significant DE genes enriched in fresh PBMCs with genes from the sampling-time signature [[4]](https://www.zotero.org/google-docs/?4DGjL4).

**Figure S2.** **FixNCut protocol tested in mouse lung samples.** (**a**) Bright-field (**left**), DAPI fluorescent (**middle**), and two microscopy images overlay (**right**) of fresh (**top**) and fixed (**bottom**) mouse lung cells. (**b**) Comparative analysis of the number of detected UMIs (**left**) and genes (**right**) for each cell barcode based on sequencing reads using a linear model. (**c**) Gene annotation categories for all captured genes in both conditions or uniquely in fresh or fixed mouse lung. (**d**) Distribution of the main QC metrics (number of UMIs, genes, and percentage of mitochondrial genes) by protocol, considering all cell types. (**e**) Distribution of the main QC metrics by cell-type and protocol. (**f**) Overlap of highly variable genes (HVGs) shared between fresh and fixed mouse lung samples, considering 3000 HVGs computed by protocol. (**g**) Linear regression model comparing average gene expression levels of expressed genes between fresh and fixed samples by cell-type. The coefficient of determination (R2) computed with Pearson correlation and the corresponding p-value are indicated. (**h**) Dotplot showing average expression for the top genes (*x-axis*) for all 20 cell types (*y-axis*). The dot size reflects the percentage of cells in a cluster expressing each gene, and the color represents the average expression level.

**Figure S3. FixNCut protocol tested in mouse colon samples.** (**a**) Comparative analysis of the number of detected UMIs (**left**) and genes (**right**) for each cell barcode based on sequencing reads using a linear model. (**b**) Gene annotation categories for all captured genes in both conditions or uniquely in fresh or fixed mouse colon. (**c**) Distribution of main QC metrics (number of UMIs, genes, and percentage of mitochondrial genes) by protocol. (**d**) Distribution of main QC metrics by cell type and protocol. (**e**) Overlap of highly variable genes (HVGs) shared between fresh and fixed mouse colon samples, considering 3000 HVGs computed for each sample independently. (**f**) Linear regression model comparing the average gene expression levels of expressed genes by cell type. The coefficient of determination (R2) computed with Pearson correlation and the corresponding p-value are indicated. (**g**) Dotplot showing average expression for the top genes (*y-axis*) for all 16 cell types (*x-axis*). The dot size reflects the percentage of cells in a cluster expressing each gene, and the color represents the average expression level.

**Figure S4.** **Long-term storage of fixed mouse lung samples.** (**a**) Comparative analysis of the number of detected UMIs (**left**) and genes (**right**) for each cell barcode based on sequencing reads using a linear model. (**b**) Gene annotation categories for all captured genes, either in all three conditions or uniquely in cryo or fixed/cryo mouse lungs. (**c**) Distribution of main QC metrics (number of UMIs, genes and percentage of mitochondrial genes) by protocol, considering all cell types. (**d**) Distribution of main QC metrics by cell-type and protocol. (**e**) Overlap of highly variable genes (HVGs) shared between protocols, considering the 3000 HVGs computed for each protocol independently. (**f**) Linear regression model comparing the average gene expression levels of expressed genes by cell-type and protocol; fixed/cryo vs fixed (**top**) and fixed/cryo vs cryo (**bottom**). The coefficient of determination (R2) computed with Pearson correlation and the corresponding p-value are indicated. (**g**) Dotplot showing average expression for the top genes (x-axis) for all 20 cell types (y-axis). Dot size reflects the percentage of cells in a cluster expressing each gene, and the color represents the average expression level.

**Figure S5. FixNCut protocol tested in human colon biopsies.** (**a**) Comparative analysis of the number of detected UMIs (**left**) and genes (**right**) for each cell barcode based on sequencing reads using a linear model. (**b**) Gene annotation categories for all captured genes, either in all conditions or uniquely in fresh, fixed, cryo or fixed/cryo human colon biopsies. (**c**) Distribution of main QC metrics (number of UMIs, genes and percentage of mitochondrial genes) by protocol. (**d**) Distribution of main QC metrics by cell-type and protocol. (**e**) Overlap of highly variable genes (HVGs) shared between protocols, considering the 3000 HVGs computed for each condition independently. (**f**) Linear regression model comparing average gene expression levels of expressed genes by cell-type and protocol; fixed vs fresh (**top**) and fixed/cryo vs cryo (**bottom**). (**g**) Dotplot showing average expression for the top genes (*x-axis*) for all 21 cell types (*y-axis*). Dot size reflects the percentage of cells in a cluster expressing each gene, and the color represents the average expression level. (**h**) Score of warm collagenase gene signature [[2]](https://www.zotero.org/google-docs/?LheyZZ) for human mouse colon by cell population across protocols.

**Figure S6. Flow cytometry analysis in fixed cells and tissues in mouse and human.** (**a**) Representative flow cytometry experiment of forward scatter (FSC) and side scatter (SSC) in cryopreserved (**left**) and cryo+fixed (**right**) human PBMCs from healthy donors (n=3). (**b**) Representative flow cytometry experiment of cell viability based on DAPI expression in cryopreserved (**left**) and cryo+fixed (**right**) human PBMCs from healthy donors (n=3). (**c**) Representative flow cytometry experiment of cell apoptosis based on the expression of Annexin V FITC and PI in fresh (**left**) and fixed (**right**) freshly isolated PBMCs. Cells were analyzed after isolation and fixation at day 0 (**top**) and 2-days after fixation (**bottom**) (n=4). (**d**) Representative gating strategy of one experiment analyzed by flow cytometry of freshly isolated PBMCs from healthy donors (n=4). PBMCs were stained with anti-human CD3, CD19, CD4 and CD8 monoclonal antibodies (mAbs). T cells were selected by the positive expression of CD3. CD4 positive and CD8 positive T cells were selected from CD3 positive. (**e**) Representative flow cytometry experiment of forward scatter (FSC) and side scatter (SSC) (**top**) and cell viability based on DAPI expression (**bottom**) of human colon biopsies from IBD donors.
