# Supplementary figures and images for: FixNCut: single-cell genomics through reversible tissue fixation and dissociation
Source: Genome Biol. 2024 Mar 29;25:81. doi: 10.1186/s13059-024-03219-5 (PMC10979608; doi:10.1186/s13059-024-03219-5)

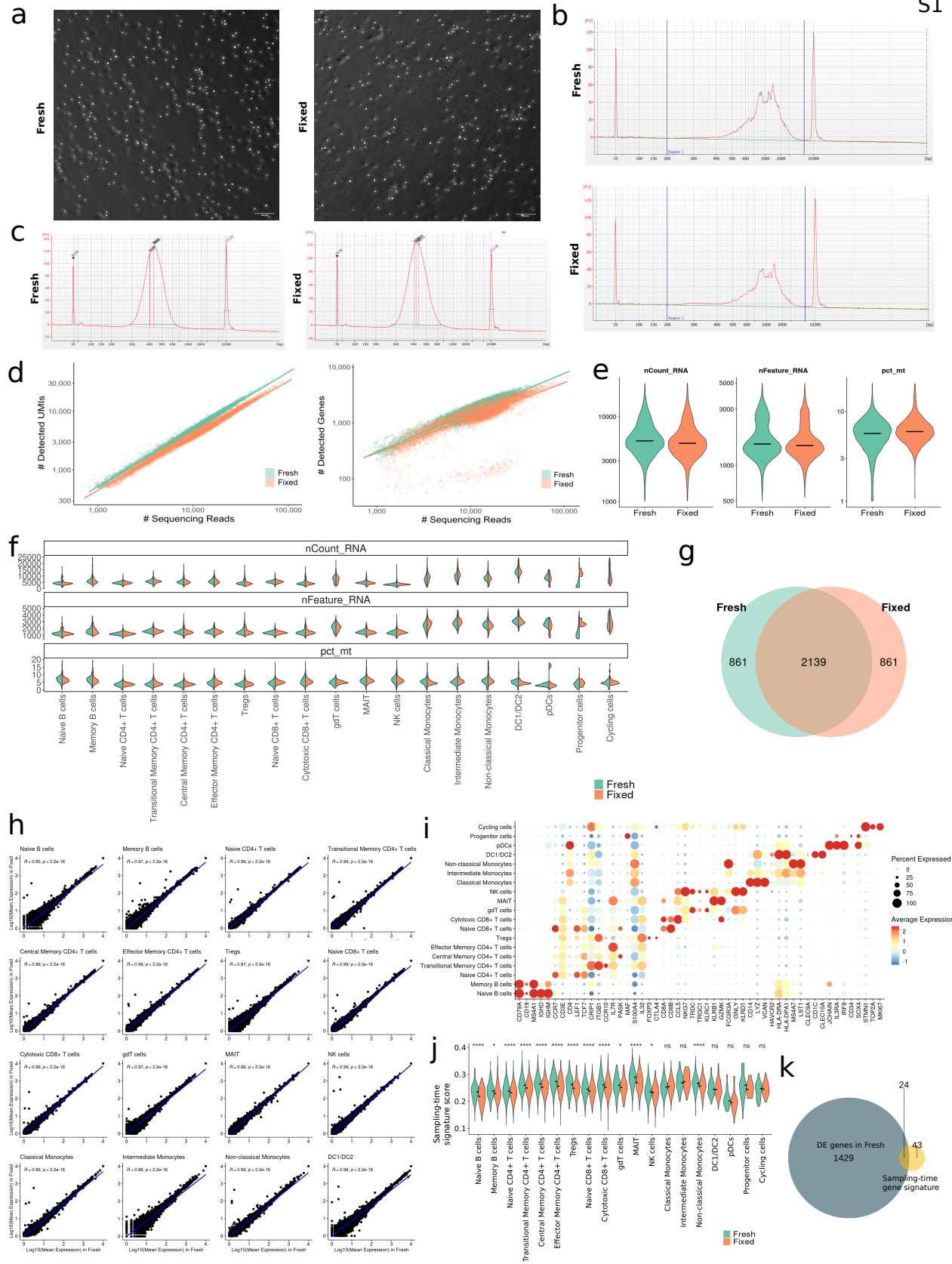

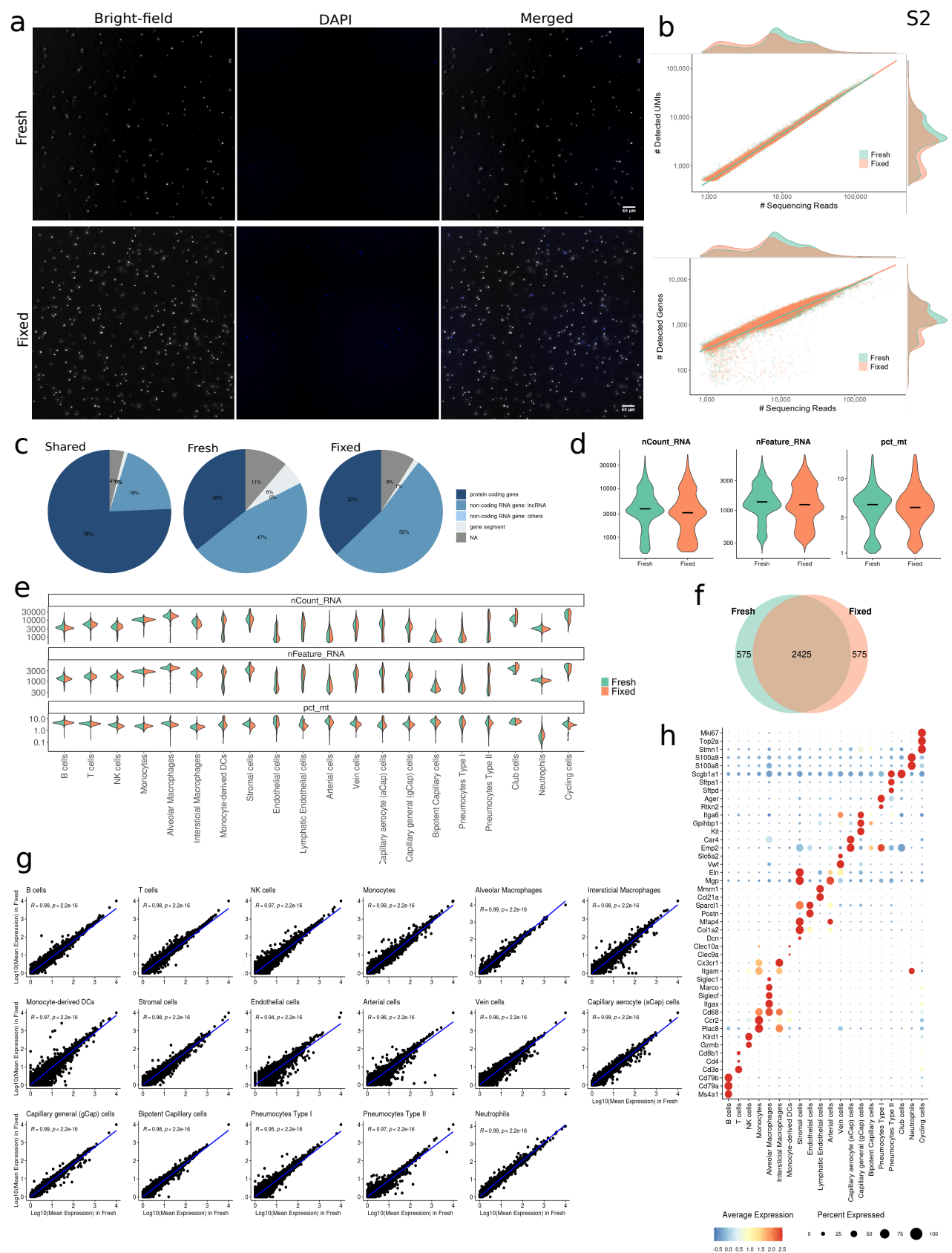

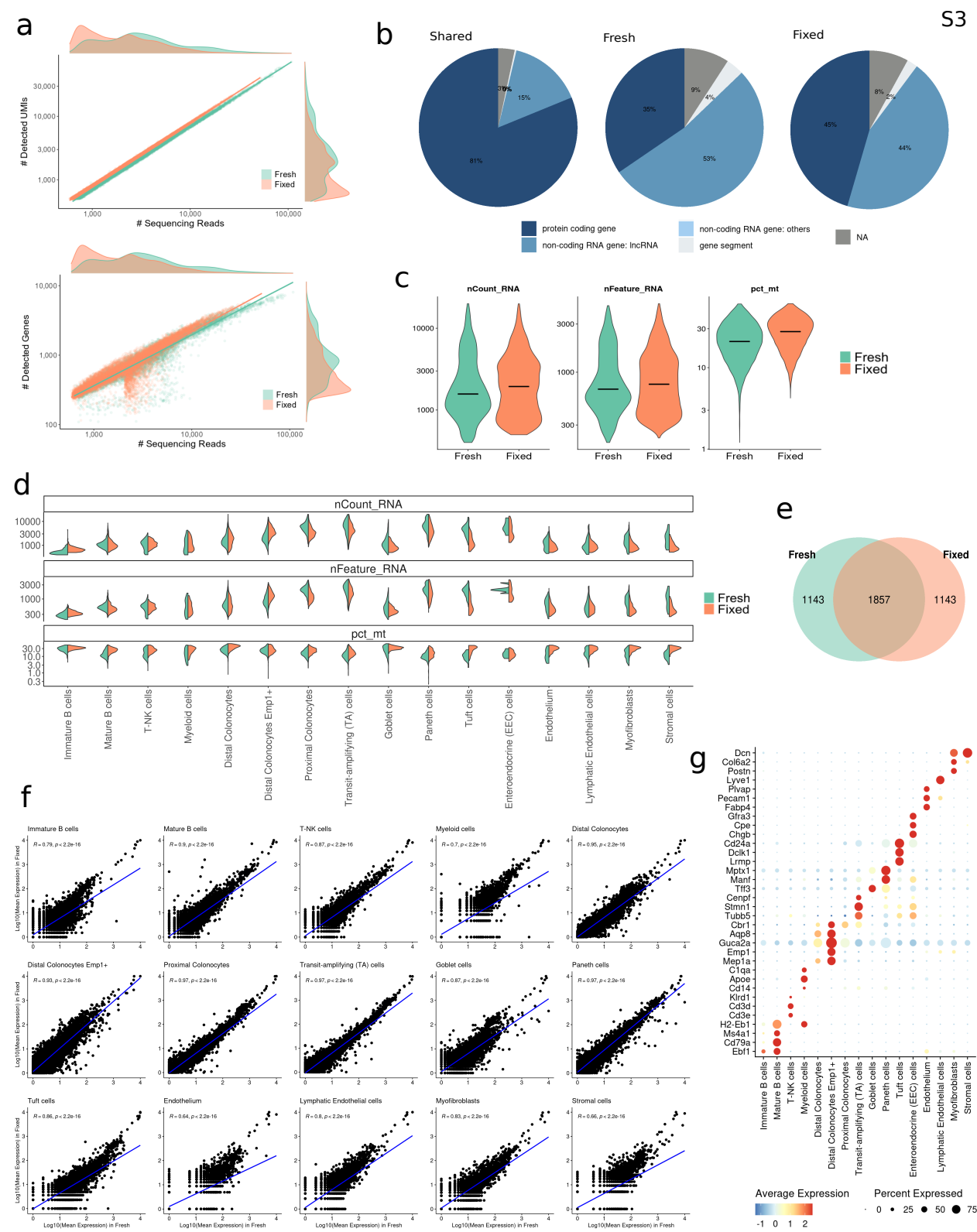

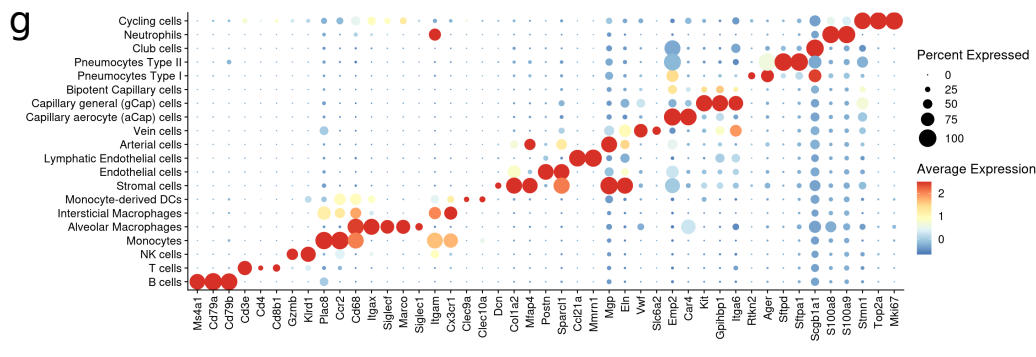

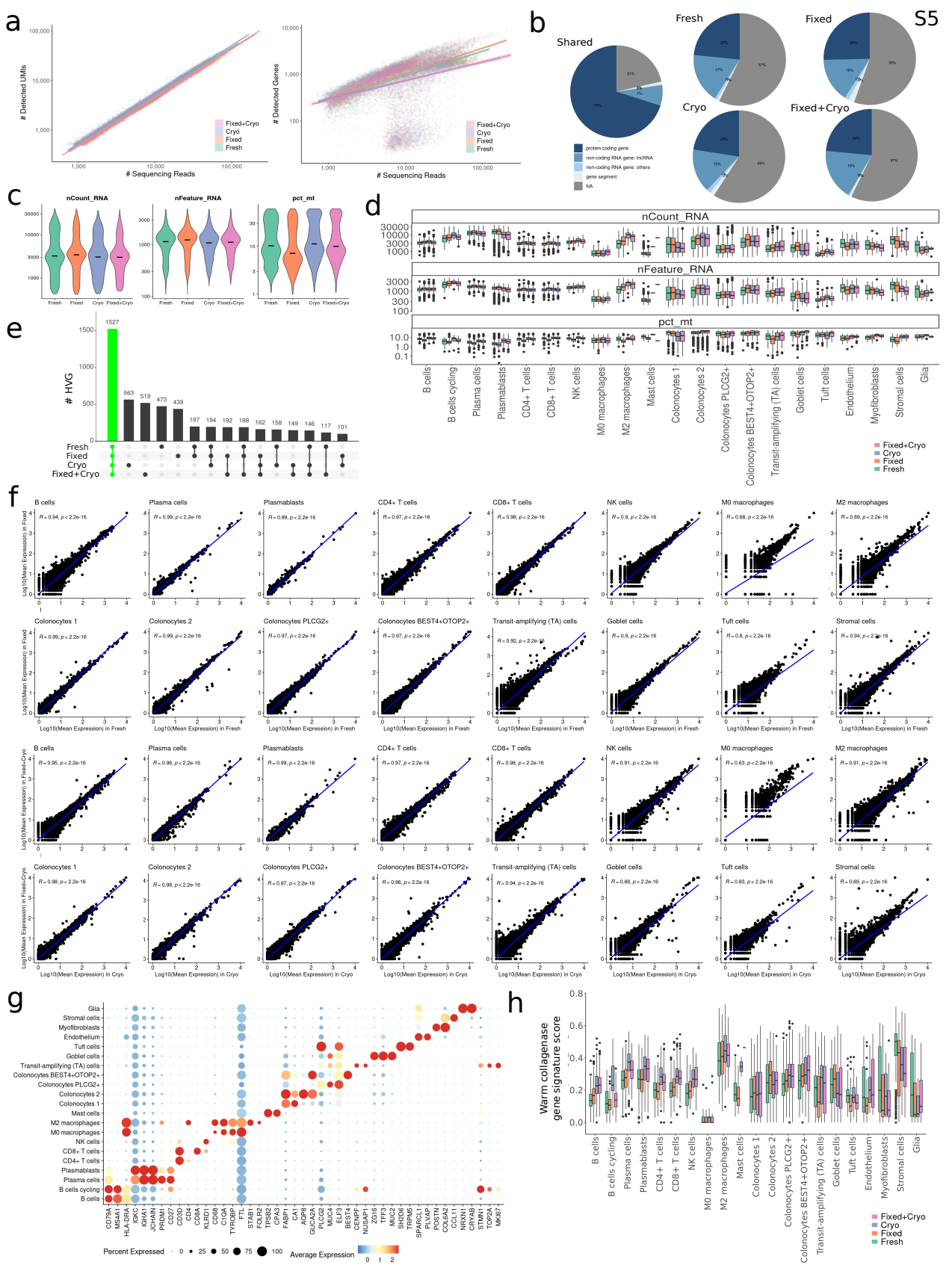

a

Cryopreserved

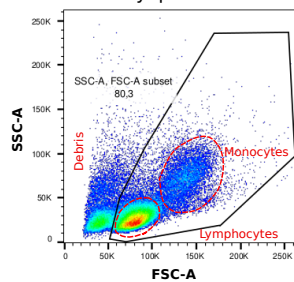

Cryo + Fixed

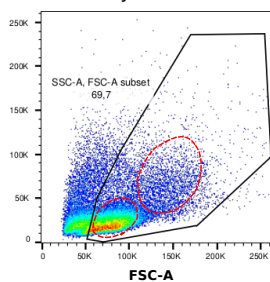

b

Cryopreserved

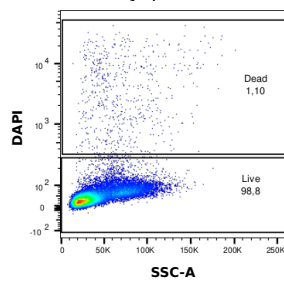

Cryo + Fixed

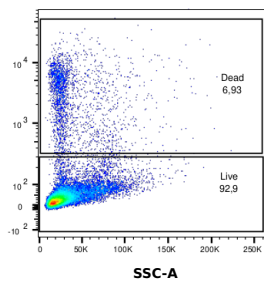

c

Fresh

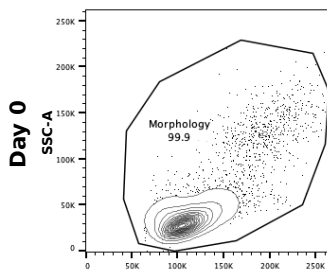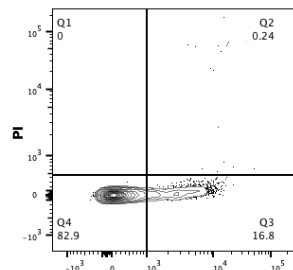

Fixed

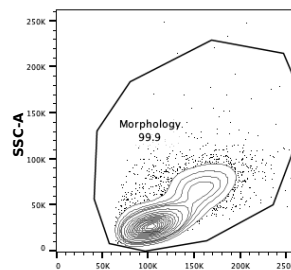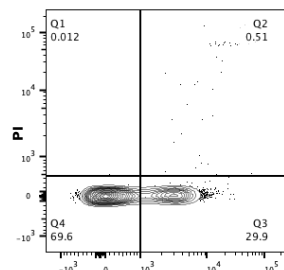

Day 2

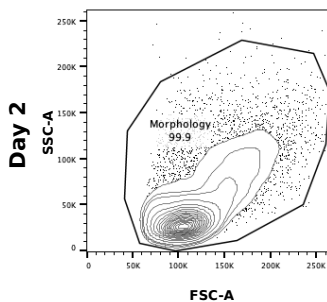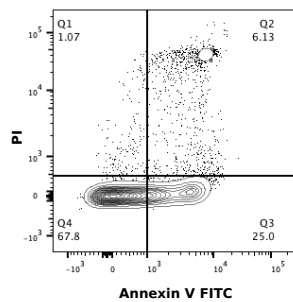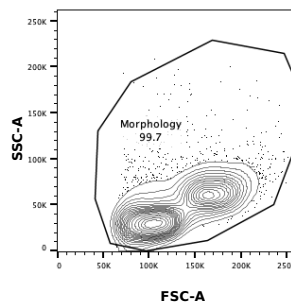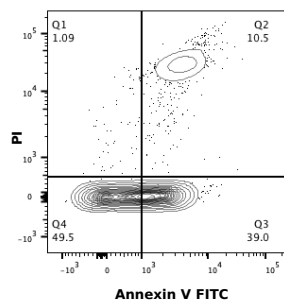

d

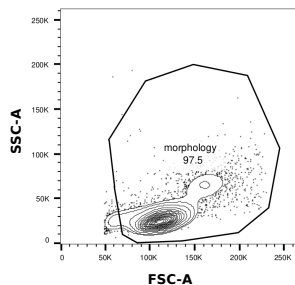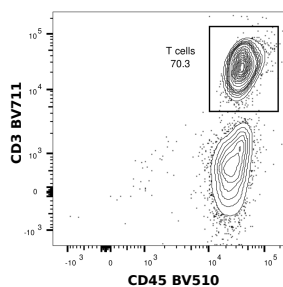

Gated on T cells

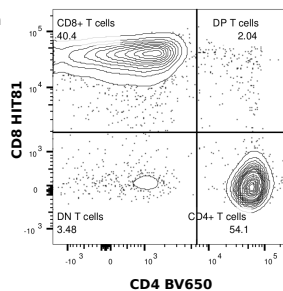

e

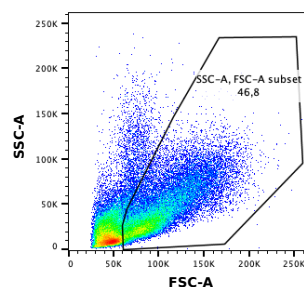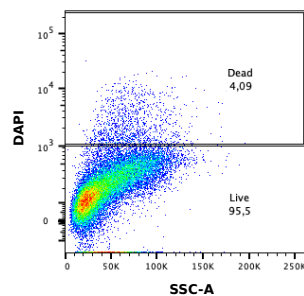

Supplement: Supplementary file 1 — Additional file 1: Supplementary Figures. Supplementary Figures 1-6 and Supplementary figure legends. [file 13059_2024_3219_MOESM1_ESM.zip › additional files figures docx and pdf/FixNCut_AdditionalFile_SuppFigs.pdf]
