# Supplementary material for: FixNCut: single-cell genomics through reversible tissue fixation and dissociation
Source: Genome Biol. 2024 Mar 29;25:81. doi: 10.1186/s13059-024-03219-5 (PMC10979608; doi:10.1186/s13059-024-03219-5)
Supplement: Supplementary file 16 — Additional file 16. Peer review history. [file 13059_2024_3219_MOESM16_ESM.docx]

**Review history**

**First round of review**

**Reviewer 1**

The authors describe FixNCut, a new single-cell genomics sample preparation workflow that involves first fixing samples with DSP prior to dissociation (a very good idea) in order to avoid dissociation artifacts before reversing the fixation and performing scRNA-seq. Overall, I find the manuscript to be a significant contribution to the field. However, I have a few points of concern which generally relate to (1) method reproducibility and (2) claims that might be overly broad. The experiments/analyses in the manuscript are as follows:

Figure 1. Comparing fresh and fixed PBMCs to demonstrate that DSP fixation (1) does not lead to significant decrease in sample quality at the level of detected cell states and cell type proportions and (2) minimizes the influence of known sample preparation artifacts - Experiments and analyses were extremely thorough and well-executed.

* Major comments: None
* Minor comments:
1. The claim that "T lymphocytes appeared to be particular affected" by sample handling is a bit shaky. The authors report a single p-value… which T-cell populations does this apply to? All of them? Is it an average? Also, most of the listed genes linked to the sampling handling signature (e.g., NFKBIA, CXCR4, JUND, and JUN) are particularly expressed by fresh vs fixed MAITs and this pattern is not maintained in other T-cell populations. It is worth being careful with this claim because some of these genes (particularly CXCR4) are very important in T-cell biology.

Figure 2. Comparing fresh vs fixed mouse lung tissue - Good experimental design but significant issues with data analysis.

* Major comments:
1. The authors claim that FixNCut enhances gene capture efficiency (both at level of detected number of genes and UMIs), but the analyses are unconvincing. No statistical tests are performed to assess whether the observed increase in median mean expression from 0.0244 to 0.0314 in fresh vs fixed cells is significant. Also, if FixNCut increases gene capture efficiency, wouldn't the fixed-specific genes be higher than the fresh-specific genes? Also, couldn't this observation be explained by differences in the sup (e.g., ambient transcripts, RBCs as was seen in the PBMC experiments, etc.) and/or differences in doublet formation rates (authors are quite vague about how they handled doublet detection for this data in the Methods)? Finally, no statistical tests are used to determine whether the difference in UMI detection is significant, and any effect in detected genes and UMIs seems to be driven by fixed rep1. Lots of issues with this analysis - needs to be refined in revised manuscript.
2. "Importantly, after filtering out low-quality cells and neutrophils, the main QC metrics in fixed samples showed more consistent distributions…" Why is it appropriate to remove neutrophils? Neutrophil drop out is less of an issue in mouse data relative to human sample preps, and they appear to have similar RNA content and detected gene numbers as B and AT1 cells - the major difference appears to be that neutrophils weirdly dropped out in fixed rep1 (also monocytes…).
3. Based on the statement "The absence of batch-effects linked to replicates or protocols was demonstrated by the PCA and UMAP representations," the authors claim that there are no observed batch effects between fresh and fixed lungs (although B and T cells do appear different)… but then also claim that fixation leads to improved gene detection and differences in cell type proportions. Which one is it? Based on the premise of the method, aren't batch effects for fresh vs fixed a 'good' thing, especially when those batch effects are due to reduced stress?

* Minor comments:
1. Fig. S2b: "fixed lung samples showed more detected genes in both replicates, mostly lncRNAs, indicating higher library complexities" - Authors do not show the preponderance of lncRNAs anywhere in this figure or in Fig. 2. Also, authors may want to comment on why this would be observed here but not in the PBMC data.
2. Canonical lung tissue-resident macrophages are alveolar and interstitial macrophages. The authors identify alveolar macs but not interstitial… are these the Apoe+ macs? They should adjust their naming to better reflect the literature.
3. The authors use Fig. 2F to claim that "high correlation across gene programs supported the absence of alterations in major biological processes" except there are clear instances where the samples cluster according to preparation condition instead of lung (e.g., apoptosis, UPR, inflammation). The logic of how to interpret Fig. 4f is confusing.
4. The fixed replicates appear highly reproducible in the reads-x-gene plot in Fig. 2B while the fresh samples appear discordant in this plot. When parsing down to single cells, the opposite is true - fresh samples appear very similar, whereas fixed samples have divergent trendlines. What explains this?

Experiment 3. Comparing fresh and fixed mouse colon tissue showing that FixNCut results in increased detection of sensitive colonic epithelial cell populations - Good experimental design and data analysis.

* Major comments:
1. No other issues except for the need to rule out alternative hypotheses for explaining the increase in gene detection (e.g., if the improved capture efficiency is demonstrated by the higher exonic read mapping fraction, then why isn't this also observed in the lung data?) - this analysis workflow was much cleaner than lung analysis.

* Minor comments:
1. Per major comment #3 above, I am unsure what point the authors are trying to convey with the Harmony analysis - if FixNCut is having a noticeable effect on how cells handle tissue dissociation then you should expect batch effects, no?

Figure 4. Comparing cryopreserved, fixed, and fixed+cryopreserved mouse lungs to demonstrate future applicability for complex experimental designs - Good experimental design and data analysis.

* Major comments:
1. No other issues except the purpose of excluding neutrophils from analyses needs to be better justified.
* Minor comments: None.

Figure 5. Quantifying gene expression signatures related to sample preparation in the lung and colon datasets - solid analyses that need a bit more refinement.

* Major comments:
1. The authors gloss over the fact that, while FixNCut does indeed lead to decreased dissociation-induced signatures in non-immune lung stromal cells (and the observation of bimodality is especially interesting), FixNCut seems to increase these signatures in immune cells. When considered along with the prior observations about variable immune cell sampling after fixation, is it possible that fixation efficiency is cell type specific (as is observed in combinatorial indexing applications)?
2. I believe the authors forgot to refer to a main text/supplemental figure to support the statement "sampling-time signatures, originally defined for PBMCs, showed a significant decrease upon fixation."
3. Authors should also assess the extent to which stress-signature genes are also differentially expressed by the fixed samples - For example, in Fig. 5C it looks like ~50% of the stress-signature genes are also fresh DEGs… are the other ~50% fixed DEGs? I imagine not, but this needs to be shown to make the point the authors are trying to make. Same concept holds for the analyses shown in Fig. 5F and Fig. 5I.

* Minor comments:
1. Add statistics to Fig. 5H violin plots (maybe as a p-value heatmap?)

Figure 6. Comparing fresh, cryopreserved, fixed, and fixed+cryopreserved primary human samples to demonstrate applicability to clinical samples.

* Major comments:
1. The changes in captured immune cell proportions are seemingly all over the place between these different experiments - e.g., here, the preserving effect of fixation on B-cell proportions is quite clear, but then this effect is not observed in any of the other datasets. Seems like this is an important point for the authors to explicitly address, as it seems like fixation may be introducing its own set of artifacts into single-cell genomics sample prep workflows.
* Minor comments: None.

Figure 7. Assessing compatibility of FixNCut with antibody and LMO labeling and imaging.

* Major comments: None.
* Minor comments: None.
1. Should add legends for the flow plots.

General minor comments:
* Authors neglect to add citations when it seems relevant, for example:
1. Intro: "In this regard, preservation methods have been developed that enable sample collection and storage, expanding the applications of single cell sequencing to personalized medicine and collaborative research" - authors cite these methods.
2. 2nd results section discussing logistical challenges with clinical trials: "Here, sampling artifacts, including biases in gene expression and cell type composition, are frequently observed in fragile solid tissue types" - this is probably true, and FixNCut is still useful even if it isn't, but the authors have to either provide the appropriate reference or lighten the claim
* In the introduction, the authors state "Cross-linking fixatives, such as formaldehyde and paraformaldehyde (PFA), are used with specialized single-cell assays, but are incompatible with commonly applied high-throughput single-cell RNA sequencing (scRNA-seq) protocols. Moreover, formaldehyde- based fixation generally impedes the application of sample multiplexing…" but the 10x fix cell kit uses PFA and you can multiplex at the level of probe set indices. Still a good point of comparison to raise, but the authors could include a little bit more nuance when introducing the differences to avoid reader confusion.
* Methods: Table 4, 'conjugate' column for LMO rows says Alexa-647, which I assume is a typo (unless you used fluorophore-conjugated LMOs)
* PCA plot axes should be "PC1" and "PC2" not "PCA1" and "PCA2"
* Add plot titles and lymphoid and myloid gate labels to Fig. S6a and Fig. S6b to improve interpretation - immunology-focused readers will know that myeloid cells are normally shifted in FSCxSSC plots, but authors shouldn't expect all readers to know this.
* Discussion: Authors may want to temper claims about applicability with ATAC-seq and multiome when they haven't tested whether FixNCut is applicable with downstream nuclei isolation and transposition.

On balance, I think that FixNCut represents a significant advance for the single-cell genomics field and is an extremely promising approach. However, the limits of the method need to be better explored, and the bioinformatics analyses presented here need to be refined. Thus, I believe that this manuscript should be conditionally-accepted for publication in Genome Biology assuming that the major comments are adequately addressed in the revised manuscript.

**Reviewer 2**

Jimenez Gracia and colleagues introduce FixNCut, a pioneering methodology designed for the early fixation of samples before cell dissociation in single-cell transcriptomics. Their journey begins with the realization that existing live dissociation protocols subject cells to stress and are impractical for clinical and similar scenarios where the sampling, dissociation, and subsequent processing must occur in different locations, often on separate days. ue has been extensively discussed in the literature, and various approaches have been proposed. Nevertheless, each individual approach faces limitations in its broader applicability.

Many existing fixative solutions involve fixation after dissociation, which still exposes dissociated cells to stress effects. In response, the authors have developed their own method for fixation and dissociation. The key observation, in my view, is the use of DSP, a fixative previously used in the literature, to first fix the cells and then dissociate them. Consequently, cells in the dissociation process are already fixed, preventing changes in their gene expression patterns.

The authors initially compare the fixative capabilities and metrics obtained after fixation in cells that do not require dissociation, such as PBMCs. These analyses reveal that the quality control metrics are highly similar, although fresh samples exhibit a stress-related signature linked to live processing. Subsequently, the authors explore the application of FixNCut to solid mouse tissues, including lung and colon samples. After examining the effects of long term preservation, the authors go on to show the validity of FixNCut in human clinical tumor samples. Finally, the authors show that FixNCut samples can still be labeled with antibodies and that they can be embedded in paraffin for spatial proteomics.

This manuscript introduces a groundbreaking method that, if widely adopted, promises to revolutionize the field of single-cell transcriptomics. The ability to fix tissue before cell dissociation is a key innovation, allowing for the collection of a large number of samples. Furthermore, it enables the preservation of clinical samples for single-cell transcriptomics and opens doors to various other research applications.

The manuscript contains several areas that could be strengthened in a revised version. To begin, the authors dedicate limited attention to the fixation process itself, primarily focusing on the single-cell transcriptomic results it yields. There is a relative absence of comprehensive information regarding the cytometric, microscopic, molecular, and cellular characteristics of the fixed samples, aside from their single-cell transcriptomic properties. Moreover, the authors mention the necessity of mincing the tissue before applying FixNCut, but they do not provide a clear rationale for this requirement, nor do they seem to have thoroughly investigated this aspect. A more thorough examination of this issue and an explanation of its necessity would enhance the manuscript's overall clarity and completeness. Lastly, the authors concentrate exclusively on processing samples with the commercial platform 10X Genomics, neglecting the existence of other options. Demonstrating the applicability of FixNCut to alternative methods or, at the very least, acknowledging and discussing these options would not only expand the scope of the manuscript but also contribute to a more comprehensive exploration of its potential. Addressing these concerns would significantly bolster the manuscript and position it as a strong candidate for publication in Genome Biology.


Major comments
FixNCut cellular, cytometric, microscopic, molecular and cellular properties:
While some of the cytometric details can be seen later in Figure 7 and associated supplementary material, I would naively think that this is key to the introduction of FixNCut as a novel method. The microscopy images provided are of limited resolution, and it is difficult to address if there are any differences. Likely the authors do not want to repeat the whole experiment, but it would be beneficial to show higher resolution microscopy images of the FixNCut cells compared to fresh. I also think that it would be good to know if the numbers of cells obtained (from comparable, or even matched samples) after dissociation in fresh and FixNCut dissociations are comparable.

Mincing of the tissue
The authors mince the tissue and it naively makes sense, as one would not think that DSP would penetrate large chunks of tissue in a split second, and this could introduce biases in gene expression as the reagent penetrates. However, the cutting of live dissected tissue can also (and surely will) induce stress responses. Then, the results of the authors are limited by this aspect, and they show that they FixNCut samples do not have stress responses when compared to fresh. The question then is: should the dissected tissue be thrown directly in trizol and compared to a matched samples where the dissection is minced, then dissociated and then thrown to trizol, would there be stress differences? This is a possibility that could limit the validity and the significance of the method. I do not think that the authors should do these experiments, as their method clearly addresses a large amount of the stress responses associated to dissociation if not all. But the authors could at least elaborate on why they chose to design the protocol as it is presented, and offer information on the tissue penetration properties of the FixNCut reagent.

Applicability to other platforms
The authors stick to 10X Genomics, but other single cell transcriptomic platforms are available and the authors could at the very least comment on the suitability, from a theoretical point of view, of FixNCut for these other platforms. Some platforms have their own fixation reagents and it would be good to be provided with some thoughts on the possibilities of FixNCut to work or not on these.

Minor comments
Page 5
Lines 28-30
DSP has been used to preserve cells for single cell transcriptomics. The authors could stress the differences between these previous uses and their FixNCut protocol? Were these previous uses done on cells that were already dissociated?
Lines 50-51
"Fixed cells showed highly similar morphology compared to fresh PBMCs in bright-field microscopy, with no evident changes in cell phenotypes or sample clumping after fixation (Fig. S1a)". The images provided by the authors do not reveal much of the morphology of the cells. This similarly applies to the images presented in Figure S2a

Page 6
Lines 27-29
"All cell types and states were found across both protocols at similar proportions, except for classical monocytes and NK cells, which showed small but significant differences, being slightly increased in fresh and fixed, respectively (Fig. 1h)"
What do the authors make of these differences? Which one is closer to microscopy data or other countings of the PBMC population?
Lines 37-41
"Importantly, we observed a reduction in technical artifacts introduced during sample processing prior to single-cell experiments upon fixation. Specifically, gene expression alterations previously defined to correlate with ex vivo PBMC handling (18) showed a significant reduction upon fixation (p < 2.2e-16) (Fig. 1k). Moreover, a sampling-time gene signature obtained from single-cell benchmarking studies (4) also showed a significant reduction in the fixed PBMCs (p < 2.2e-16) (Fig. 1j). Interestingly, T lymphocytes appeared to be particularly affected (p <= 0.0001), showing the strongest protection from sampling artifacts in fixed cells (Fig. S1j)"
It is unclear to me why these scores are shown as a violin plot (j) and a UMAP plot (k). Also, while the score is visibly higher in j, and the authors provide a significance test, it is unclear where can the differences be seen in the UMAP. Why do the authors show it? Can this be replaced by another violin plot, which would show the distribution of the score better? If the authors want to show where the score is high, perhaps they can plot the violin plots by cluster?

Page7
Lines 4-7
The authors could elaborate on the why they need to mince the tissue. Did they try to fix whole organs? What prevents this?
Lines 13-15
"Notably, at the same sequencing depth, fixed lung samples showed more detected genes in both replicates, mostly lncRNAs, indicating higher library complexities (Fig. S2b)"
Where do the authors show that the detected genes are mostly lncRNAs?
Lines 29-31
"The absence of batch-effects linked to replicates or protocols was demonstrated by the PCA and UMAP representations (Fig. 2d; Fig. S2g)"
how do the umaps and PC plots demonstrate the absence of batch effects? The UMAP shows areas where the fresh and fixed samples are distributed differently. Same for the first 2 PCs, and if the authors scroll through the remaining PCs they might find some that separates fresh and fixed. Interpreting low dimensional projections of such highly dimensional data is highly problematic. As I understand from the methods, this analysis does not have any integration algorithm, the authors might want to highlight that.
Lines 36-46
The differences in cell proportions are considerable. Similar to Figure 1h, what do the authors make of these differences? There are differences rather pronounced between the fixed replicates, which could point to differences in sample preparation.
Page 8
Lines 7-9
"Our results indicate that FixNCut provides several benefits, including improved transcriptome capture accuracy, as evidenced by a higher number of total reads mapped to the reference and a higher exonic fraction (Fig. 3a)"
How do the authors explain this higher exonic fraction? That was not the case with lung
Lines 16-18
"Further, we identified batch-effects associated with the sample preparation protocol as shown in both PCA and UMAP representations, which were removed by sample integration (Fig. 3d; Fig. S3e)"
Do the authors think that these batch effects are due to the fixation or just the different 10X channels in use?
Lines 44-45
While the authors describe their cryopreservation process in the methods, it would be useful to the reader to be told here very briefly.
Page 9
Lines 37-38 and 43-45
"Interestingly, the signature scores for these populations displayed a bimodal-like distribution, indicating an uneven effect within cell populations (Fig. 5b)"
Please comment on that. While the fresh is overall very clearly higher in some clusters, there are some clusters where the fixed has a higher score, like T cells, NK cells, etc. Surely the authors can give add some comments. This can be done together with the statement related to the colon samples
Page 10
Lines 26-29
Why spatial proteomics? The authors could elaborate and further comment about the use of FixNCut in spatial transcriptomics.
Also on this section, it seems to me that this is an important result that the authors could further describe. There are details in the methods but I think that a reader would like to see here some essential definition in one sentence of the paraffin embedding process.

**Authors’ response to reviewers**

Reviewer #1
The authors describe FixNCut, a new single-cell genomics sample preparation workflow that involves first fixing samples with DSP prior to dissociation (a very good idea) in order to avoid dissociation artifacts before reversing the fixation and performing scRNA-seq. Overall, I find the manuscript to be a significant contribution to the field. However, I have a few points of concern which generally relate to (1) method reproducibility and (2) claims that might be overly broad. The experiments/analyses in the manuscript are as follows:

Figure 1.
Comparing fresh and fixed PBMCs to demonstrate that DSP fixation (1) does not lead to significant decrease in sample quality at the level of detected cell states and cell type proportions and (2) minimizes the influence of known sample preparation artifacts - Experiments and analyses were extremely thorough and well-executed.
* Minor comments:
1. The claim that "T lymphocytes appeared to be particularly affected" by sample handling is a bit shaky. The authors report a single p-value… which T-cell populations does this apply to? All of them? Is it an average?
Thank you for your attention to detail. We want to clarify that we computed and reported the p-value for each individual population of T cells (obtained by comparing the score distribution of each T cell population), as indicated in the Fig. S1j. All T cell subtypes exhibited a significant p-value (p<=0.0001), except for gdT cells, which showed a significant but larger p-value (p<=0.05). To improve clarity, we have provided a more detailed breakdown of the p-value for the respective T cell subpopulations in the main text.
Also, most of the listed genes linked to the sampling handling signature (e.g., NFKBIA, CXCR4, JUND, and JUN) are particularly expressed by fresh vs fixed MAITs and this pattern is not maintained in other T-cell populations. It is worth being careful with this claim because some of these genes (particularly CXCR4) are very important in T-cell biology.
We appreciate the reviewer’s feedback and agree that it is important to consider that different cell populations may express genes at varying levels due to their intrinsic biology. To address this concern, we computed the sampling-time gene signature from Massoni-Badosa et al. (1) for each cell and evaluated the score distribution between fixed and fresh samples (Fig. 1j), but also stratified by cell subpopulations (Fig. 1k and Fig. S1j). This approach aimed at ensuring that differences observed between fresh and fixed samples within a cell type could be attributed to DSP-fixation rather than to differences in compositional and gene expression levels of subpopulations.
When examining gene expression levels from the sampling-time signature (as presented in Fig. 1l), the dotplot reveals, irrespective of the scaled expression levels across populations, that all genes are expressed at lower levels in fixed PBMCs compared to fresh samples across all cell types. However, the reviewer rightfully emphasized the implications of reporting a subset of genes as main contributors (e.g. CXCR4 in MAIT cells is highly expressed as compared to other T cell populations). Considering the primary objective of the paper to introduce a new sampling method, rather than to delineate single genes associated with sampling-time effects, we have decided to exclude claims about specific genes to avoid misleading conclusions. The manuscript text has been modified accordingly.

Figure 2.
Comparing fresh vs fixed mouse lung tissue - Good experimental design but significant issues with data analysis.
We greatly appreciate the attention that both reviewers have given to the analysis of mouse lung tissue, especially in relation to the data analysis where the neutrophil population was excluded. We have carefully reviewed this section and we will outline the reasons for excluding neutrophils in the original manuscript version. However, we decided to address this aspect in the revised manuscript by keeping neutrophils in the analysis and by excluding Experiment 1, where mice were not perfused. Below and in the respective comments, we provide additional considerations to support our decision.
As detailed in the manuscript’s methods, the lung samples from Experiment 1 were not subjected to perfusion. Upon analyzing these results, we observed a substantial presence of neutrophils, especially in the fresh sample, that could stem from both circulating or tissue-resident neutrophils (2). Consequently, we chose to repeat this experiment (Experiment 2), sacrificing mice that were perfused prior to sample collection. Here, the detecting neutrophils suggests the presence of a tissue-associated neutrophil population, with fresh and fixed samples showing comparable relative cell numbers. Considering these results, we initially decided to exclude neutrophils from all samples for downstream analysis, with the intention to rescue the first experiment in the original manuscript. However, we acknowledge both reviewer's concern that excluding neutrophils removes an important tissue-resident cell type and impedes a comprehensive analysis. We agree that the primary scope of the manuscript is evaluating the performance of FixNCut comprehensively across cell types and, hence, changed the strategy to including neutrophils while excluding Experiment 1 and non-perfused mice.

* Major comments:
1. The authors claim that FixNCut enhances gene capture efficiency (both at the level of detected number of genes and UMIs), but the analyses are unconvincing. No statistical tests are performed to assess whether the observed increase in median mean expression from 0.0244 to 0.0314 in fresh vs fixed cells is significant. Also, if FixNCut increases gene capture efficiency, wouldn't the fixed-specific genes be higher than the fresh-specific genes?
We appreciate the reviewer’s observation. Although no statistical proof was included in the manuscript, we conducted a statistical analysis to compare the distribution of gene expression levels between fixed (Mean Expression, ME=0.0314) and fresh (ME=0.0244) samples, considering both replicates. Specifically, we performed a one-sided Wilcoxon rank sum test (alternative: greater), obtaining a p-value = 1.449e-12, confirming that genes in the fixed condition were significatively highly expressed globally compared to the fresh sample.
We now repeated the same analysis considering only Experiment 2 (perfused lung samples). Although the mean gene expression was still larger for the fixed compared to the fresh sample (ME=0.024 versus ME=0.023), the one-sided Wilcoxon rank sum test (alternative: greater) yielded a p-value = 0.2778, pointing to no significant differences in gene expression levels between both conditions. Given this new scenario, we found it reasonable to exclude this section of the results from the revised manuscript, as it does not provide any additional information.
Also, couldn't this observation be explained by differences in the sup (e.g., ambient transcripts, RBCs as was seen in the PBMC experiments, etc.) and/or differences in doublet formation rates (authors are quite vague about how they handled doublet detection for this data in the Methods)?
We thank the reviewer for bringing attention to the absence of information concerning doublet handling. While it was not explicitly outlined in the previous methods version, we did take doublets into account in our analysis. In brief, we ran Scrublet (3) to predict doublets in all libraries and retained this information for consideration and evaluation in the subsequent analysis steps. A complete description of the details regarding doublet calling and other relevant considerations have been incorporated in the Methods section in the revised manuscript version.
Finally, no statistical tests are used to determine whether the difference in UMI detection is significant, and any effect in detected genes and UMIs seems to be driven by fixed rep1. Lots of issues with this analysis - needs to be refined in revised manuscript.
We appreciate the reviewer’s concern regarding the absence of statistical tests when comparing the differences in library complexity based on the captured genes and UMIs across different library sequencing depths. We assessed captured genes and UMIs at different sequencing depths, randomly sampled from the complete library size, with steps of 5-10 million reads. Following the reviewer’s suggestion, we considered various options to evaluate differences in library complexity along sequencing depth between protocols. First, we applied a Mann-Whitney U test, to compare values at specific points along the curves. Second, we fit a regression model to each curve to compare the coefficients, slopes or other parameters to assess significant differences. For both approaches, we conducted a comprehensive analysis for all comparisons of this manuscript (human PBMCs, mouse lungs and colon, long-term storage, and human colon biopsies). The results from both approaches agreed in all the comparisons and we included only the latter approach (overlapping of confidence intervals for the independent parameter) in the revised manuscript version. Please refer to the revised text and Methods section for a full description of the results and statistics, respectively. In brief, we found no statistically significant differences in the number of detected UMIs and genes in any of the comparisons, except for the long-term storage using mouse lungs. Here, fixed/cryo samples exhibited a statistically significant lower number of UMIs compared to cryo-only samples. However, this difference did not impact the number of recovered genes, which showed no statistical difference.

2. "Importantly, after filtering out low-quality cells and neutrophils, the main QC metrics in fixed samples showed more consistent distributions…" Why is it appropriate to remove neutrophils? Neutrophil drop out is less of an issue in mouse data relative to human sample preps, and they appear to have similar RNA content and detected gene numbers as B and AT1 cells - the major difference appears to be that neutrophils weirdly dropped out in fixed rep1 (also monocytes…).
We thank the reviewer for his comment. As outlined above, considering the technical differences retrieving the lung samples between both experiments, we initially opted to exclude neutrophils to enhance the comparability of both experiments. However, we acknowledged the reviewer’s concern that homeostatic, infiltrating neutrophils are present in lungs and should not be excluded. Thus, we chose to exclude experiment 1 (non-perfused lungs) in the revised version of the manuscript and maintained the complete lung cellular composition, including neutrophils.

3. Based on the statement "The absence of batch-effects linked to replicates or protocols was demonstrated by the PCA and UMAP representations," the authors claim that there are no observed batch effects between fresh and fixed lungs (although B and T cells do appear different)… but then also claim that fixation leads to improved gene detection and differences in cell type proportions. Which one is it? Based on the premise of the method, aren't batch effects for fresh vs fixed a 'good' thing, especially when those batch effects are due to reduced stress?
We thank the reviewer for providing these thoughtful comments. In reference to the PCA and UMAP analysis presented for fresh and fixed mouse lungs, it is important to highlight that we did not employ any data integration at this point. As correctly highlighted by the reviewer, the observed differences were very subtle, with cell type-defining transcriptomes showing notably stronger differences than those associated with cellular stress (attenuated with DSP-fixation protocol). Thus, the activation of stressed genes did not significantly impact the overall transcriptome profile, though stress may present challenges in retrieving specific cell states. This became particularly evident when delving into a more granular exploration (i.e. stress signatures and DEGs) of cell types and states across conditions.

* Minor comments:
1. Fig. S2b: "fixed lung samples showed more detected genes in both replicates, mostly lncRNAs, indicating higher library complexities" - Authors do not show the preponderance of lncRNAs anywhere in this figure or in Fig. 2. Also, authors may want to comment on why this would be observed here but not in the PBMC data.
We thank the reviewer for this comment. Firstly, we wish to clarify that while we have not presented a panel displaying the results on which our observations are based, we did conducted a thorough analysis utilizing the complete list of gene annotations sourced from the Mouse Genome Informatics website (https://www.informatics.jax.org). Secondly, it is important to note that this analysis was specifically carried out in mouse lungs. To provide further support to our previous observations, we have repeated the analysis for the mouse lung tissue (Experiment 2), but also mouse colon tissue as well as for human colon biopsies. For the human samples, we utilized the comprehensive list of gene annotations obtained from the HUGO Gene Nomenclature Committee (HGNC) (https://www.genenames.org/).
The mouse lung tissue, after excluding non-perfused samples (Experiment 1), exhibited results similar to the previously described. As stated in the revised manuscript, DSP-fixation showed the greatest improvement when applied to more sensitive tissues, such the mouse colon biopsies. For long term-storage on mouse lung samples, we found a similar gene annotation profile of captured genes across conditions. In human colon biopsies, we identified a large number of genes present in all conditions, primarily consisting of protein-coding genes while genes exclusively captured in a specific condition were mostly non-annotated genes. These new results have been included in the manuscript main text as Supplementary Figures S2c, S3b, S4b and S5b respectively.
For human PBMCs, we performed the same analysis; however, we did not include the results in the manuscript since the main objective of the project focuses on solid tissues. In this case, we identified a large number of genes present in both fresh and fixed conditions (n=22,481), primarily consisting of protein-coding genes (66%), followed by non-annotated genes (26%). Conversely, genes exclusively captured in either fresh or fixed conditions are mostly non-annotated genes (50% and 53%, respectively), with a one-third of protein-coding genes in both conditions (33% and 30%, respectively).

2. Canonical lung tissue-resident macrophages are alveolar and interstitial macrophages. The authors identify alveolar macs but not interstitial… are these the Apoe+ macs? They should adjust their naming to better reflect the literature.
We thank the reviewer for his input. As mentioned, murine lung tissue-resident macrophages (Cd68+) could be present in the alveolus (i.e. alveolar macrophages, AlvMϕs) or in the interstitium (interstitial macrophages, IntMϕs). As described by Bain and MacDonald and illustrated in their Fig. 1, murine AlvMϕs express high CD11c (Itgax), SiglecF, MARCO and CD169 (Siglec1) while lacking CD11b (Itgam) expression. In contrast, murine IntMϕs are characterized by high levels of CD11b (Itgam) coupled with a lack of SiglecF expression and the presence of other genes, such as Cx3cr1 (4). We agree that in our original manuscript only described the alveolar macrophages and a second population, referred to as macrophages Apoe+. After a detailed comparison of the differential expressed genes for each lung macrophage population, we confirmed that Apoe+ macrophages indeed represent the interstitial macrophage profile. With the aim of harmonizing cell population names with the available literature, we have now revised the manuscript accordingly and updated the text and images.

3. The authors use Fig. 2F to claim that "high correlation across gene programs supported the absence of alterations in major biological processes" except there are clear instances where the samples cluster according to preparation condition instead of lung (e.g., apoptosis, UPR, inflammation). The logic of how to interpret Fig. 4f is confusing.
We appreciate the reviewer's observations. The authors acknowledge that the presented results may have been misleading due to the inclusion of both mouse lung replicates, perfused and non-perfused tissue. However, it is important to emphasize that, in all instances, we were dealing with significant and very robust correlations (R2 > 0.95) across all conditions. However, the lack of lung perfusion (Experiment 1) might have been the primary factor contributing to lower correlations and discordances. Specifically, fresh non-perfused lungs captured a substantially higher number of neutrophils compared to fixed (and Experiment 2). Given that the biological processes were evaluated across all populations, compositional changes could have led to reduced gene profile correlation. As discussed above, we retained only perfused lung biopsies and repeated the full analysis, which we are confident has further enhanced the quality of the manuscript.

4. The fixed replicates appear highly reproducible in the reads-x-gene plot in Fig. 2B while the fresh samples appear discordant in this plot. When parsing down to single cells, the opposite is true - fresh samples appear very similar, whereas fixed samples have divergent trendlines. What explains this?
We thank the reviewer for his observation. It is crucial to emphasize that while Fig. 2b illustrates the genes captured based on sequencing depth (reads) considering the entire library, Fig. S2 plots depict the captured genes and UMIs per cell, without employing any downsampling. Additionally, it is essential to note that these results were derived from both experiments (perfused and non-perfused mouse lungs), which will have led to distinct population proportions (given the compositional nature of the data). Hence, the distribution of sequenced reads across all cell types will have been disproportionately distributed across cell types, an effect that could only be observed at the cell level. After excluding Experiment 1 and for a more comprehensive analysis of library complexity across conditions, we expanded the previous Fig. 2b by including additional plots illustrating the number of captured UMIs by sequencing depth (reads), in addition to the number of captured genes. Furthermore, we have incorporated statistical analysis to assess differences in library complexity between conditions.

Figure 3.
Comparing fresh and fixed mouse colon tissue showing that FixNCut results in increased detection of sensitive colonic epithelial cell populations - Good experimental design and data analysis.
* Major comments:
1. No other issues except for the need to rule out alternative hypotheses for explaining the increase in gene detection (e.g., if the improved capture efficiency is demonstrated by the higher exonic read mapping fraction, then why isn't this also observed in the lung data?) - this analysis workflow was much cleaner than lung analysis.
We value the reviewer's observation, a comment also that was also raised by Reviewer #2. We agree on the significance of gaining a better understanding of the reasons behind the differences in the percentage of reads mapping to exonic regions and the total number of captured genes (which has been shown not to be statistically significant though). In response, we conducted additional statistical analysis delving deeper into the library composition. Specifically, we quantified the total number of captured UMIs, which offers an additional layer of information about library complexity to disentangle the relationship between exonic reads and the total number of detected genes. This new analysis has been conducted for all comparisons (human PBMCs, mouse lung and colon, and human colon biopsies) and has been included in the main text of the manuscript in Figures panels 1b, 2b, 3b, 4b, and 6b.
It is important to note that having higher numbers of UMIs does not always translate to higher gene counts if genes are represented by multiple UMIs. Accordingly, the fixed mouse colon sample recovered a higher percentage of reads mapped to exonic regions, a similar number of UMIs, but a higher number of detected genes compared to the fresh sample. A plausible explanation is that the fixed sample captures more mature transcripts, supported by more exonic reads, resulting in a higher number of detected genes. Conversely, in the fresh sample, there may be a larger number of transcripts captured belonging to the same genes. This pattern is akin to what was observed for human PBMCs, where, despite the fixed sample recovering a lower percentage of reads mapped to exonic regions and a lower number of UMIs, it ultimately captures a similar total number of genes. After excluding non-perfused lung experiments, the mouse lung comparison (fixed vs. fresh) showed comparable values for all the assessed library complexity metrics.

* Minor comments:
1. Per major comment #3 above, I am unsure what point the authors are trying to convey with the Harmony analysis - if FixNCut is having a noticeable effect on how cells handle tissue dissociation then you should expect batch effects, no?
We thank the reviewer for his comment regarding batch effects and integration. We concur with the reviewer’s insight that enhancements in single-cell outcomes, attributed to the utilization of the FixNCut protocol in comparison to fresh biopsies (e.g. capturing additional genes, improving gene expression levels, or avoiding stress-related genes, as evident in our results), contribute to differences in the transcriptomic profile when projecting cells into the 2D space using UMAP. Nevertheless, as one of our primary objectives was to compare cell composition and gene expression profiles by cell population, and considering that the overall cellular profile remains intact albeit with a decrease in cell quality, we employed integration with the Harmony package to mitigate technical differences and to annotate cells collectively. Recognizing the significance of clarity on this aspect, we have revised the manuscript accordingly to provide a clear message to the reader.

Figure 4.
Comparing cryopreserved, fixed, and fixed+cryopreserved mouse lungs to demonstrate future applicability for complex experimental designs - Good experimental design and data analysis.
* Major comments:
1. No other issues except the purpose of excluding neutrophils from analyses needs to be better justified.
We greatly appreciate the attention that both reviewers have given to the analysis of mouse lung tissue, especially in relation to the data analysis where the entire neutrophil population was excluded. It is essential to note that the assessment of FixNCut coupled with cryopreservation for long-term storage was conducted solely with samples from the mice used in the Replicate 2, where lungs were perfused. We concurred with the reviewer that retaining the full tissue cell composition profile is important. In the revised version of the manuscript, we have retained the neutrophil population (as done as well in Fig. 2). We believe this decision, coupled with the updated results after a full re-analysis, will offer a reliable and clear interpretation on the benefits of DSP-fixation.

Figure 5.
Quantifying gene expression signatures related to sample preparation in the lung and colon datasets - solid analyses that need a bit more refinement.
* Major comments:
1. The authors gloss over the fact that, while FixNCut does indeed lead to decreased dissociation-induced signatures in non-immune lung stromal cells (and the observation of bimodality is especially interesting), FixNCut seems to increase these signatures in immune cells. When considered along with the prior observations about variable immune cell sampling after fixation, is it possible that fixation efficiency is cell type specific (as is observed in combinatorial indexing applications)?
Thank you for highlighting the important observation regarding the differential impact of the FixNCut method on immune versus non-immune lung stromal cells, particularly in relation to dissociation-induced signatures. Your point about the increased signatures in immune cells, in contrast to decreased signatures in non-immune cells, is a crucial aspect of our findings. It is important to note that the fixation process using FixNCut is very quick. Any fixation-induced stress responses would require a longer time frame to be adequately expressed at the mRNA level than possible during the fixation process. Therefore, the dissociation-induced signatures observed, particularly in immune cells, are unlikely to be a direct consequence of the fixation process itself.
The differential signatures in immune cells could be attributed to multiple factors, including pre-existing stress conditions in the cells before fixation. One hypothesis we consider is that the observed signatures in immune cells may reflect stressed cells that were present prior to fixation, possibly due to stressors encountered after tissue resection. In fresh tissues, these stressed cells may dissipate or not survive long enough to be detected. In contrast, in fixed tissues, the fixation process essentially ‘freezes’ the cells in their current state, including any stress responses, allowing for their detection in the fixed samples, but not in the parallel fresh samples. While this hypothesis is compelling, exploring it in depth would require a separate line of investigation, focusing specifically on the pre-fixation stress status of different cell types and how this is captured differently in fresh versus fixed samples. However, this falls beyond the scope of our current study, which focuses on the efficacy of the FixNCut method in preserving cell integrity and minimizing dissociation artifacts.

2. I believe the authors forgot to refer to a main text/supplemental figure to support the statement "sampling-time signatures, originally defined for PBMCs, showed a significant decrease upon fixation."
We appreciate the observation, and indeed we missed to include the panel supporting this claim. After further reflexion, considering that the mentioned signature was captured on PBMCs and that the main aim of the manuscript is the application of FixNCut on tissues - for which we have evaluated dissociation-specific stress signatures (as shown in Fig.5 a,b), we finally decided not to evaluate sampling-time PBMC and therefore, we excluded this statement to prevent potential confusion and to enhance coherence of our manuscript.

3. Authors should also assess the extent to which stress-signature genes are also differentially expressed by the fixed samples - For example, in Fig. 5C it looks like ~50% of the stress-signature genes are also fresh DEGs… are the other ~50% fixed DEGs? I imagine not, but this needs to be shown to make the point the authors are trying to make. Same concept holds for the analyses shown in Fig. 5F and Fig. 5I.
We agree that a more comprehensive examination should have been conducted in this regard. We now compared the gene overlap between differentially expressed genes (DEG) identified after sample processing using a specific protocol with various stress-related gene signatures.
As illustrated in Fig. 5a, the fixed sample exhibited a similar gene signature score for the three stress-related signatures compared to the fresh sample (with only a significant difference in one gene signature). In this context, we observed an overlap of DEG for both fixed and fresh samples with the genes associated with the examined signatures. Notably, the fixed sample showed a larger overlap of genes. When evaluating mouse colon tissue or mouse lung long-term storage, we found a completely different result. In these particular situations, both fresh and cryo-only showed not only significantly higher scores for all the stress-related gene signatures, but also the largest overlapping of DEG with the stress-related gene signatures. Fixed and fixed+cryo samples, in contrast, exhibited minimal overlap with stress-related genes. Altogether, it can be asserted that the more pronounced the stress impact during sample collection, processing or storage, the more favorable the results provided by DSP-fixation. We have now modified the text to accurately reflect this fact and avoid any potential misleading interpretations.
These new results have been included in the manuscript in Figure 5 as Panels C, F and I. We replaced previous Venn diagrams of DEGs with UpsetPlot. This new representation includes DEG in all the conditions under study: fresh, fixed, cryo and fixed/cryo.

* Minor comments:
1. Add statistics to Fig. 5H violin plots (maybe as a p-value heatmap?)
We thank the reviewer for highlighting the absence of statistical information and suggesting the use of a heatmap instead of violin plots with added p-values. Taking into account that the fixed lung sample had already been examined for stress-related signatures in comparison with the fresh biopsy (Fig. 5a-c), we have contemplated excluding the fixed condition from Fig. 5h-i. Thus, in the current version of the manuscript, we exclusively compared cryo-only and fixed/cryo since, at this stage, the focus is on evaluating putative stress-related to dissociation for long-term storage. Furthermore, this decision was made with the intention of preserving the ViolinPlot panel including p-values (instead of a heatmap), as it is crucial for observing the bimodal distribution present in certain cryo-only cell populations compared to fixed/cryo. In addition, this choice also aligned with the goal of maintaining consistency throughout the manuscript figure.

Figure 6.
Comparing fresh, cryopreserved, fixed, and fixed+cryopreserved primary human samples to demonstrate applicability to clinical samples.
* Major comments:
1. The changes in captured immune cell proportions are seemingly all over the place between these different experiments - e.g., here, the preserving effect of fixation on B-cell proportions is quite clear, but then this effect is not observed in any of the other datasets. Seems like this is an important point for the authors to explicitly address, as it seems like fixation may be introducing its own set of artifacts into single-cell genomics sample prep workflows.
We appreciate the reviewer’s observation. While we have investigated the effect of DSP-fixation in both mouse and human species, it is crucial to note that major cell types, as studied and described here, are present across all species (and the DSP-fixative does not differentiate between human, mouse or other species). However, the population proportions in each case may vary. This variability, coupled with the fact that human colon biopsies are derived from a diseased patient, could be a factor influencing whether observed differences become statistically significant or not, introducing an added layer of complexity in the analysis. In conclusion, we acknowledge that endothelial, epithelial and stromal population proportions are largely recovered in the DSP-fixed condition, offering a more accurate representation of the tissue’s cellular composition, as supported by several studies (5,6). However, it is essential to recognize that cell population proportion data is compositional, implying that any decrease/increase in one cell population influences the proportion of other populations.

Figure 7.
Assessing compatibility of FixNCut with antibody and LMO labeling and imaging.
* Minor comments:
1. Should add legends for the flow plots.
We appreciate the reviewer for bringing this detail to our attention and included the legend.

General minor comments:
* Authors neglect to add citations when it seems relevant, for example:
1. Intro: "In this regard, preservation methods have been developed that enable sample collection and storage, expanding the applications of single cell sequencing to personalized medicine and collaborative research" - authors cite these methods.
We appreciate the reviewer for his comment. We would like to highlight that these citations, along with their respective methods’ pros and cons, were thoroughly discussed later in the manuscript and, therefore, were not included at this juncture.

2. 2nd results section discussing logistical challenges with clinical trials: "Here, sampling artifacts, including biases in gene expression and cell type composition, are frequently observed in fragile solid tissue types" - this is probably true, and FixNCut is still useful even if it isn't, but the authors have to either provide the appropriate reference or lighten the claim.
We thank the reviewer for his valuable comment. Arceneaux et al. demonstrated that DSP-fixation of colonic epithelium, compared to using fresh samples, ensures the preservation of larger RNA content while reducing ambient contamination, particularly in fragile cell types such as secretory and absorptive cells, which may be prone to cell death during dissociation steps (5). Furthermore, Habib et al. proposed an alternative method known as Div-Seq, employing single-nucleus instead of single-cell, to capture and study cell types such as neurons. This approach is particularly relevant in organs or tissues, such as the Central Nervous System, where standard dissociation protocols may lead to the disruption of the cytoplasmic context and the loss of specific cell types due to enzymatic and mechanical forces (6). To support our claim regarding the biases in cellular composition and gene expression in fragile solid tissues, we have included these references and updated the manuscript accordingly. Moreover, our ongoing efforts optimizing and applying the FixNCut protocol to other organs and tissue types either fragile tissues, such as pancreas, or rare ones like bone, for which none or very little scRNA-seq articles have been published so far; however, these specific applications are not part of our ongoing efforts for this manuscript.

* In the introduction, the authors state "Cross-linking fixatives, such as formaldehyde and paraformaldehyde (PFA), are used with specialized single-cell assays, but are incompatible with commonly applied high-throughput single-cell RNA sequencing (scRNA-seq) protocols. Moreover, formaldehyde-based fixation generally impedes the application of sample multiplexing…" but the 10x fix cell kit uses PFA and you can multiplex at the level of probe set indices. Still a good point of comparison to raise, but the authors could include a little bit more nuance when introducing the differences to avoid reader confusion. The reviewer correctly notes the advancements in 10x Genomics' new fixative kit and multiplexing capabilities. However, the key advantage of DSP-fixed samples over formalin-fixed samples lies in their compatibility with standard polyA-based expression measurement. Crucially, because DSP does not cross-links RNA, the transcript integrity is not affected in DSP-fixed samples and are comparable to fresh samples (7). This means that all techniques applicable to fresh-frozen samples are potentially feasible with DSP-fixed samples as well, even though our manuscript centers on 3' approaches. However, from ongoing projects, we have gathered evidence that DSP-fixed samples are not only compatible with 3' end sequencing, but also with other techniques like 5' end sequencing (e.g. in this paper the authors also apply DSP to capture T-cell receptors (TCR) on PBMC or T cells (8)), ATAC-seq, and Cut&Tag. This level of compatibility extends beyond what is possible with formalin-fixed samples. In summary, DSP fixation offers a versatile platform that maintains the integrity and diversity of transcriptomic and genomic analyses, comparable to fresh samples, which is a significant step forward in fixed-sample analysis.

* Methods: Table 4, 'conjugate' column for LMO rows says Alexa-647, which I assume is a typo (unless you used fluorophore-conjugated LMOs)
We appreciate the reviewer for spotting the error in Table 4. Indeed, the ‘conjugate’ column for LMO rows stating Alexa-647 should not be there, and has been corrected.

* PCA plot axes should be "PC1" and "PC2" not "PCA1" and "PCA2"
Thank you for noticing the typo error with “PCA” instead of “PC”. The axes on the PCA plots have been corrected.

* Add plot titles and lymphoid and myeloid gate labels to Fig. S6a and Fig. S6b to improve interpretation - immunology-focused readers will know that myeloid cells are normally shifted in FSCxSSC plots, but authors shouldn't expect all readers to know this.
We thank the reviewer’s suggestion. We have included plot titles and gate labels (lymphoid, myeloid and debris) to facilitate the interpretation of results for our readers.

* Discussion: Authors may want to temper claims about applicability with ATAC-seq and multiome when they haven't tested whether FixNCut is applicable with downstream nuclei isolation and transposition.
We appreciate the reviewer's suggestion and have revised/removed respective sections in the Discussion.


Reviewer #2
Jimenez Gracia and colleagues introduce FixNCut, a pioneering methodology designed for the early fixation of samples before cell dissociation in single-cell transcriptomics. Their journey begins with the realization that existing live dissociation protocols subject cells to stress and are impractical for clinical and similar scenarios where the sampling, dissociation, and subsequent processing must occur in different locations, often on separate days. It has been extensively discussed in the literature, and various approaches have been proposed. Nevertheless, each individual approach faces limitations in its broader applicability.
Many existing fixative solutions involve fixation after dissociation, which still exposes dissociated cells to stress effects. In response, the authors have developed their own method for fixation and dissociation. The key observation, in my view, is the use of DSP, a fixative previously used in the literature, to first fix the cells and then dissociate them. Consequently, cells in the dissociation process are already fixed, preventing changes in their gene expression patterns.
The authors initially compare the fixative capabilities and metrics obtained after fixation in cells that do not require dissociation, such as PBMCs. These analyses reveal that the quality control metrics are highly similar, although fresh samples exhibit a stress-related signature linked to live processing. Subsequently, the authors explore the application of FixNCut to solid mouse tissues, including lung and colon samples. After examining the effects of long term preservation, the authors go on to show the validity of FixNCut in human clinical tumor samples. Finally, the authors show that FixNCut samples can still be labeled with antibodies and that they can be embedded in paraffin for spatial proteomics.
This manuscript introduces a groundbreaking method that, if widely adopted, promises to revolutionize the field of single-cell transcriptomics. The ability to fix tissue before cell dissociation is a key innovation, allowing for the collection of a large number of samples. Furthermore, it enables the preservation of clinical samples for single-cell transcriptomics and opens doors to various other research applications.
The manuscript contains several areas that could be strengthened in a revised version. To begin, the authors dedicate limited attention to the fixation process itself, primarily focusing on the single-cell transcriptomic results it yields. There is a relative absence of comprehensive information regarding the cytometric, microscopic, molecular, and cellular characteristics of the fixed samples, aside from their single-cell transcriptomic properties. Moreover, the authors mention the necessity of mincing the tissue before applying FixNCut, but they do not provide a clear rationale for this requirement, nor do they seem to have thoroughly investigated this aspect. A more thorough examination of this issue and an explanation of its necessity would enhance the manuscript's overall clarity and completeness. Lastly, the authors concentrate exclusively on processing samples with the commercial platform 10X Genomics, neglecting the existence of other options. Demonstrating the applicability of FixNCut to alternative methods or, at the very least, acknowledging and discussing these options would not only expand the scope of the manuscript but also contribute to a more comprehensive exploration of its potential. Addressing these concerns would significantly bolster the manuscript and position it as a strong candidate for publication in Genome Biology.

Major comments
FixNCut cellular, cytometric, microscopic, molecular and cellular properties: While some of the cytometric details can be seen later in Figure 7 and associated supplementary material, I would naively think that this is key to the introduction of FixNCut as a novel method.
We thank the reviewer’s observation. It is important to emphasize that the primary focus of our article is to demonstrate the application of FixNCut for solid tissues using single-cell genomics technologies. However, given the current expansion of applications in the single-cell genomics field, such as CITE-seq or sample multiplexing, which involve cell staining, we found it relevant to include a proof-of-concept and feasibility validation as outlook for future application of the protocol. Therefore, we made the decision to showcase FixNCut’s compatibility with antibody and LMO staining through cytometry to highlight its feasibility and potential. Indeed, ongoing projects have provided evidence that DSP-fixed samples are not only compatible with standard scRNA-seq assays, but also with techniques that involve cell labeling.
The microscopy images provided are of limited resolution, and it is difficult to address if there are any differences. Likely the authors do not want to repeat the whole experiment, but it would be beneficial to show higher resolution microscopy images of the FixNCut cells compared to fresh.
We appreciate the reviewer’s observation. Based on the original images, we have now included higher resolution images. We hope that these improvements address the concern.
I also think that it would be good to know if the numbers of cells obtained (from comparable, or even matched samples) after dissociation in fresh and FixNCut dissociations are comparable.
We appreciate the reviewer’s comment. We have not conducted a comprehensive measurement of the post-dissociation cell number recovered in both fresh and fixed samples, as the primary focus in the manuscript has been on the recovery of cell composition and gene expression profile. However, thus far, we have not observed a significant or noticeable decrease in cell yield in fixed samples compared to fresh ones.
Mincing of the tissue. The authors mince the tissue and it naively makes sense, as one would not think that DSP would penetrate large chunks of tissue in a split second, and this could introduce biases in gene expression as the reagent penetrates. However, the cutting of live dissected tissue can also (and surely will) induce stress responses. Then, the results of the authors are limited by this aspect, and they show that the FixNCut samples do not have stress responses when compared to fresh. The question then is: should the dissected tissue be thrown directly in Trizol and compared to a matched sample where the dissection is minced, then dissociated and then thrown to Trizol, would there be stress differences? This is a possibility that could limit the validity and the significance of the method. I do not think that the authors should do these experiments, as their method clearly addresses a large amount of the stress responses associated with dissociation if not all. But the authors could at least elaborate on why they chose to design the protocol as it is presented, and offer information on the tissue penetration properties of the FixNCut reagent.
The authors thank the reviewer for highlighting this topic. It is important for us to further clarify the approach and purpose of our fixation step.
First, cell dissociation typically occurs without nutrients and at 37°C, a condition where transcription remains active, as highlighted by Denisenko et al. (9). Our method involves triturating tissue into 2-3 mm² pieces to increase surface area exposure to the fixative, rather than mincing. The respective wording has been corrected in the revised manuscript. Trituration ensures that DSP-fixed samples are subject to stress for a shorter duration compared to fresh tissue processing, where cells are alive during dissection and dissociation. Consequently, cells in fixed samples have limited time to experience or express stress responses. Triturating samples was also included to homogenize biopsies before moving on with either the fresh sample or performing DSP-fixation in order to avoid introducing batch-effects. We revised the Method section to clarify the application of trituration.
Second, the main aim of our paper is to leverage this methodology for single-cell analysis. The reviewer's suggestion seems to pivot back towards bulk analysis, where stress signals could be diluted rather than accentuated. It is crucial to note that stress responses are not uniformly expressed across all cells, they are more prominent in specific subsets (see Fig. S1j, and Fig. 5), also evidenced in other cited studies (e.g. Denisenko et al. (9)). This uneven distribution of stress signals means they could be missed in a bulk analysis approach, underscoring the importance of our single-cell focused method for accurately detecting and studying these responses.
Applicability to other platforms. The authors stick to 10X Genomics, but other single cell transcriptomic platforms are available and the authors could at the very least comment on the suitability, from a theoretical point of view, of FixNCut for these other platforms. Some platforms have their own fixation reagents and it would be good to be provided with some thoughts on the possibilities of FixNCut to work or not on these.
The authors thank the reviewer for this comment. We have revised both the Introduction and Discussion in order to make the claim of applicability broader and beyond the application of 10x Genomics assays. In fact, we removed reference to the company to make the statements more universal. We have also included the fact that formalin fixation is being used in specific protocols and briefly discuss their applicability and specifics.

Minor comments
Page 5
Lines 28-30: DSP has been used to preserve cells for single cell transcriptomics. The authors could stress the differences between these previous uses and their FixNCut protocol? Were these previous uses done on cells that were already dissociated?
We appreciate the reviewer’s insightful comment, which prompted us to emphasize the difference between our FixNCut protocol and previous uses of DSP for single-cell transcriptomics. In the revised manuscript, we have explicitly highlighted that our protocol is intended for application to tissues before dissociation, in contrast to previous applications on cells in suspension, including cells in culture or PBMCs.
Lines 50-51. "Fixed cells showed highly similar morphology compared to fresh PBMCs in bright-field microscopy, with no evident changes in cell phenotypes or sample clumping after fixation (Fig. S1a)". The images provided by the authors do not reveal much of the morphology of the cells. This similarly applies to the images presented in Figure S2a.
We appreciate the reviewer’s observation. The issue with the low image resolution has been addressed, as detailed earlier.

Page 6
Lines 27-29. "All cell types and states were found across both protocols at similar proportions, except for classical monocytes and NK cells, which showed small but significant differences, being slightly increased in fresh and fixed, respectively (Fig. 1h)". What do the authors make of these differences? Which one is closer to microscopy data or other countings of the PBMC population?
Thank you for your comment. In alignment with the primary objective of our manuscript, which is to demonstrate the application of FixNCut for solid tissues using single-cell genomics technologies, we utilized PBMCs solely to showcase the compatibility of FixNCut with the widely used single-cell genomics technology from 10X Genomics. Given the nature of PBMCs as a fluid suspension of cells (rather than a solid tissue requiring dissociation steps prior to scRNA-seq), we did not anticipate any remarkable changes in cellular composition. The observed differences in specific immune populations might be attributed to these particular cell states, potentially being more fragile and prone to be damaged during the encapsulation process (as clearly seen for human neutrophils that are never detected in fresh human blood samples using 10X Genomics assays). We conducted additional experiments using flow cytometry, where we found no significant differences, as illustrated in Fig. 7a. However, it is important to note that the antibodies used in this experiment (anti-CD3, -CD4, -CD8, -CD19) only allowed the quantification of major lymphoid populations, including CD4+ and CD8+ T cells and B cells. Therefore, this cytometry data is not fully comparable with the scRNA-seq results discussed before.

Lines 37-41. "Importantly, we observed a reduction in technical artifacts introduced during sample processing prior to single-cell experiments upon fixation. Specifically, gene expression alterations previously defined to correlate with ex vivo PBMC handling (18) showed a significant reduction upon fixation (p < 2.2e-16) (Fig. 1k). Moreover, a sampling-time gene signature obtained from single-cell benchmarking studies (4) also showed a significant reduction in the fixed PBMCs (p < 2.2e-16) (Fig. 1j). Interestingly, T lymphocytes appeared to be particularly affected (p <= 0.0001), showing the strongest protection from sampling artifacts in fixed cells (Fig. S1j)". It is unclear to me why these scores are shown as a violin plot (j) and a UMAP plot (k). Also, while the score is visibly higher in j, and the authors provide a significance test, it is unclear where the differences can be seen in the UMAP. Why do the authors show it? Can this be replaced by another violin plot, which would show the distribution of the score better? If the authors want to show where the score is high, perhaps they can plot the violin plots by cluster?
Thank you for your attention to detail. We completely agree with the reviewer’s observation. The ViolinPlot (Fig. 1j) displays the sampling-time signature score distribution across all cells between conditions and is used for statistical analysis. The Nebulosa UMAP (Fig. 1k) provides the density of individual signature score distributions, allowing the identification of regions with higher or lower density corresponding to specific cell types and states. However, we acknowledge that relying on the Nebulosa plot to identify affected populations might be misleading. To avoid redundancy and to enhance manuscript clarity, we have decided to exclude the Nebulosa UMAP from the revised manuscript, retaining only the Violin Plot by condition. Of note, the original manuscript included the gene signature score distribution by cell population and its associated p-value (Fig. S1j).

Page7
Lines 4-7. The authors could elaborate on why they need to mince the tissue. Did they try to fix whole organs? What prevents this?
We appreciate the reviewer’s concern regarding the tissue mincing prior tissue fixation. Please refer to the above comment “Mincing of the tissue” for a detailed explanation.

Lines 13-15. "Notably, at the same sequencing depth, fixed lung samples showed more detected genes in both replicates, mostly lncRNAs, indicating higher library complexities (Fig. S2b)". Where do the authors show that the detected genes are mostly lncRNAs?
We thank the reviewer for this comment. Firstly, we wish to clarify that while we have not presented a panel displaying the results on which our observations are based, we did conducted a thorough analysis utilizing the complete list of gene annotations sourced from the Mouse Genome Informatics website (https://www.informatics.jax.org). Secondly, it is important to note that this analysis was specifically carried out in mouse lungs. To provide further support to our previous observations, we have repeated the analysis for the mouse lung tissue, but also for mouse colon tissue and human colon biopsies. For the human samples, we utilized the comprehensive list of gene annotations obtained from the HUGO Gene Nomenclature Committee (HGNC) (https://www.genenames.org/).
The mouse lung tissue, after excluding non-perfused samples (Experiment 1), exhibited results similar to the previously described. As stated in the revised manuscript, DSP-fixation showed the greatest improvement when applied to more sensitive tissues, such the mouse colon biopsies. For long term-storage on mouse lung samples, we found a similar gene annotation profile of captured genes across conditions. In human colon biopsies, we identified a large number of genes present in all conditions, primarily consisting of protein-coding genes while genes exclusively captured in a specific condition were mostly non-annotated genes. These new results have been included in the manuscript main text as Supplementary Figures S2c, S3b, S4b and S5b respectively.
For human PBMCs, we performed the same analysis; however, we did not include the results in the manuscript since the main objective of the project focuses on solid tissues. In this case, we identified a large number of genes present in both fresh and fixed conditions (n=22,481), primarily consisting of protein-coding genes (66%), followed by non-annotated genes (26%). Conversely, genes exclusively captured in either fresh or fixed conditions are mostly non-annotated genes (50% and 53%, respectively), with a one-third of protein-coding genes in both conditions (33% and 30%, respectively).

Lines 29-31. "The absence of batch-effects linked to replicates or protocols was demonstrated by the PCA and UMAP representations (Fig. 2d; Fig. S2g)" How do the UMAPs and PC plots demonstrate the absence of batch effects? The UMAP shows areas where the fresh and fixed samples are distributed differently. Same for the first 2 PCs, and if the authors scroll through the remaining PCs they might find some that separates fresh and fixed. Interpreting low dimensional projections of such highly dimensional data is highly problematic. As I understand from the methods, this analysis does not have any integration algorithm, the authors might want to highlight that.
We confirm that we did not employ any data integration. We observed more than 80% overlapping of highly variable genes (HVG) between both conditions, along with a strong transcriptomic profile correlation (R2 > 0.99). These parameters further support the interpretation of low-dimension projection (PCA/UMAP), where intermixing between conditions was globally observed. Differences between samples were subtle, with expression programs of cell type identity being stronger than those associated with sample processing. However, as explained in the subsequent sections, although showing globally conserved gene expression profiles, notable differences were observed in differentially expressed genes and stress-signatures. Please note that these analyses have been repeated in the revised manuscript, related to the following comment by the reviewer and amendment we have made to the sample selection.

Lines 36-46. The differences in cell proportions are considerable. Similar to Figure 1h, what do the authors make of these differences? There are differences rather pronounced between the fixed replicates, which could point to differences in sample preparation.
We greatly appreciate the attention that both reviewers have given to the analysis of mouse lung tissue, especially in relation to the data analysis where the neutrophil population was excluded. We have carefully reviewed this section and we will outline the reasons for excluding neutrophils in the original manuscript version. However, we decided to address this aspect in the revised manuscript by keeping neutrophils in the analysis and by excluding Experiment 1, where mice were not perfused. Below and in the respective comments, we provide additional considerations to support our decision.
As detailed in the manuscript’s methods, the lung samples from Experiment 1 were not subjected to perfusion. Upon analyzing these results, we observed a substantial presence of neutrophils, especially in the fresh sample, that could stem from both circulating or tissue-resident neutrophils (2). Consequently, we chose to repeat this experiment (Experiment 2), sacrificing mice that were perfused prior to sample collection. Here, the detecting neutrophils suggests the presence of a tissue-associated neutrophil population, with fresh and fixed samples showing comparable relative cell numbers. Considering these results, we initially decided to exclude neutrophils from all samples for downstream analysis, with the intention to rescue the first experiment in the original manuscript. However, we acknowledge both reviewer's concern that excluding neutrophils removes an important tissue-resident cell type, impedes a comprehensive analysis and includes major batch effects. The primary scope of the manuscript was evaluating the performance of FixNCut comprehensively across cell types and, hence, changed the strategy to including neutrophils while excluding Experiment 1 and non-perfused mice.
Across the different experiments, FixNCut fixation has shown an impact on cell proportions as depicted in Fig. 2h, 3h and 4h. We observed a shift in tissue cell populations, with epithelial/endothelial/stromal cell types being enriched in DSP-fixed samples, while proportions of immune cell populations decreased. Previous work on single-nuclei RNA-seq (snRNA-seq) experiments by Slyper et al., considered “gold-standard” for cellular proportions, demonstrated that epithelial cells are highly sensitive to tissue processing and prone to cause significant loss in the most aggressive conditions (10). Thus, the results of scRNA-seq on FixNCut sample composition offer a more accurate representation of what has been described using snRNA-seq.

Page 8
Lines 7-9. "Our results indicate that FixNCut provides several benefits, including improved transcriptome capture accuracy, as evidenced by a higher number of total reads mapped to the reference and a higher exonic fraction (Fig. 3a)". How do the authors explain this higher exonic fraction? That was not the case with lungs.
We value the reviewer's observation. We agree on the significance of gaining a better understanding of the reasons behind the differences in the percentage of reads mapping to exonic regions and the total number of captured genes (which has been shown not to be statistically significant though). In response, we conducted additional statistical analysis delving deeper into the library composition. Specifically, we quantified the total number of captured UMIs, which offers an additional layer of information about library complexity to disentangle the relationship between exonic reads and the total number of detected genes. This new analysis has been conducted for all comparisons (human PBMCs, mouse lung and colon, and human colon biopsies) and has been included in the main text of the manuscript in Figures panels 1b, 2b, 3b, 4b, and 6b.
It is important to note that having higher numbers of UMIs does not always translate to higher gene counts if genes are represented by multiple UMIs. Accordingly, the fixed mouse colon sample recovered a higher percentage of reads mapped to exonic regions, a similar number of UMIs, but a higher number of detected genes compared to the fresh sample. A plausible explanation is that the fixed sample captures more mature transcripts, supported by more exonic reads, resulting in a higher number of detected genes. Conversely, in the fresh sample, there may be a larger number of transcripts captured belonging to the same genes. This pattern is akin to what was observed for human PBMCs, where, despite the fixed sample recovering a lower percentage of reads mapped to exonic regions and a lower number of UMIs, it ultimately captures a similar total number of genes. After excluding non-perfused lung experiments, the mouse lung comparison (fixed vs. fresh) showed comparable values for all the assessed library complexity metrics.

Lines 16-18. "Further, we identified batch-effects associated with the sample preparation protocol as shown in both PCA and UMAP representations, which were removed by sample integration (Fig. 3d; Fig. S3e)". Do the authors think that these batch effects are due to the fixation or just the different 10X channels in use?
We thank the reviewer for the comment regarding batch effects and integration. We argue that enhancements in single-cell outcomes, attributed to the utilization of the FixNCut protocol in comparison to fresh biopsies (e.g. capturing additional genes, improving gene expression levels, or avoiding stress-related genes, as evident in our results), contribute to differences in the transcriptomic profile when projecting cells into the 2D space using UMAP. Batch effects between 10X channels are very subtle and we would not attribute differences to the distinct 10X channels (no defects or failures during the encapsulation process). Nevertheless, as one additional objective was to compare cell composition and gene expression profiles by cell population, and considering that the overall cellular profile remains intact albeit with a decrease in cell quality, we employed integration with the Harmony package to mitigate technical differences and to annotate cells collectively. Recognizing the significance of clarity on this aspect, we have revised the manuscript accordingly to provide a clear message to the reader.
Lines 44-45. While the authors describe their cryopreservation process in the methods, it would be useful to the reader to be told here very briefly.
Thank you for your valuable suggestion. In response, we have included a brief description of the cryopreservation protocol applied in this context and referred to the methods section for further details.

Page 9. Lines 37-38 and 43-45. "Interestingly, the signature scores for these populations displayed a bimodal-like distribution, indicating an uneven effect within cell populations (Fig. 5b)". Please comment on that. While the fresh is overall very clearly higher in some clusters, there are some clusters where the fixed has a higher score, like T cells, NK cells, etc. Surely the authors can add some comments. This can be done together with the statement related to the colon samples.
Thank you for your comment regarding the bimodal signature score distribution in immune cells as compared to a unique profile for non-immune cells, which is indeed intriguing. We have not specifically tested the efficacy of fixation across individual cell types in our study. However, based on the principles of how Lomant’s reagent (DSP) works, we can provide some insights. Lomant’s reagent is a crosslinker that primarily targets amine groups in proteins, leading to the formation of stable covalent bonds. This process helps to preserve cellular and tissue architecture by ‘fixing’ proteins in place. Regarding membrane permeability, DSP is known for its ability to permeate cell membranes efficiently. This characteristic is crucial for ensuring that the reagent can effectively reach and crosslink intracellular proteins. Nevertheless, it’s important to note that DSP’s efficiency can be influenced by the density and composition of proteins on the cell surface, which might differ between immune and non-immune cells.
As for the stress signature observed in our study, we believe it is activated before the fixation process and is not a consequence of differential fixation efficiency. This activation is likely due to the physiological state of the cells prior to fixation, rather than the fixation method itself. It is worth noting that while DSP is generally considered effective for a wide range of cell types, there might be subtle variations in its efficacy across different populations. This is an area that certainly warrants further investigation, and we appreciate your suggestion to explore this aspect in future studies.

Page 10. Lines 26-29. Why spatial proteomics? The authors could elaborate and further comment about the use of FixNCut in spatial transcriptomics. Also in this section, it seems to me that this is an important result that the authors could further describe. There are details in the methods but I think that a reader would like to see here some essential definition in one sentence of the paraffin embedding process.
As suggested by the reviewer, we have expanded the results obtained from the prostate cancer biopsy and the analysis of the Phenocycler instrument (Akoya Biosciences). Ongoing collaborative efforts have since broadened the scope of DSP applications to include platforms such as Xenium, CosMx, Visium, and STereoseq; the latter being the most promising. Nevertheless, these developments are not a part of separate publications and their applications go beyond the scope of the presented manuscript. The main focus of this study was the ability to fix tissue before dissociation to reduce stress artifacts. Nevertheless, we included an outlook of future applicability in the Results and Discussion section.

References

1. Massoni-Badosa R, Iacono G, Moutinho C, Kulis M, Palau N, Marchese D, et al. Sampling time-dependent artifacts in single-cell genomics studies. Genome Biol. 2020 May 11;21(1):112.
2. Casanova-Acebes M, Nicolás-Ávila JA, Li JL, García-Silva S, Balachander A, Rubio-Ponce A, et al. Neutrophils instruct homeostatic and pathological states in naive tissues. J Exp Med. 2018 Oct 3;215(11):2778–95.
3. Wolock SL, Lopez R, Klein AM. Scrublet: Computational Identification of Cell Doublets in Single-Cell Transcriptomic Data. Cell Syst. 2019 Apr;8(4):281-291.e9.
4. Bain CC, MacDonald AS. The impact of the lung environment on macrophage development, activation and function: diversity in the face of adversity. Mucosal Immunol. 2022 Feb;15(2):223–34.
5. Arceneaux D, Chen Z, Simmons AJ, Heiser CN, Southard-Smith AN, Brenan MJ, et al. A contamination focused approach for optimizing the single-cell RNA-seq experiment. iScience. 2023 Jun 29;26(7):107242.
6. Habib N, Li Y, Heidenreich M, Swiech L, Avraham-Davidi I, Trombetta JJ, et al. Div-Seq: Single-nucleus RNA-Seq reveals dynamics of rare adult newborn neurons. Science. 2016 Aug 26;353(6302):925–8.
7. Attar M, Sharma E, Li S, Bryer C, Cubitt L, Broxholme J, et al. A practical solution for preserving single cells for RNA sequencing. Sci Rep. 2018 Feb 1;8(1):2151.
8. Nesterenko PA, McLaughlin J, Cheng D, Bangayan NJ, Burton Sojo G, Seet CS, et al. Droplet-based mRNA sequencing of fixed and permeabilized cells by CLInt-seq allows for antigen-specific TCR cloning. Proc Natl Acad Sci. 2021 Jan 19;118(3):e2021190118.
9. Denisenko E, Guo BB, Jones M, Hou R, de Kock L, Lassmann T, et al. Systematic assessment of tissue dissociation and storage biases in single-cell and single-nucleus RNA-seq workflows. Genome Biol. 2020 Jun 2;21(1):130.
10. Slyper M, Porter CBM, Ashenberg O, Waldman J, Drokhlyansky E, Wakiro I, et al. A single-cell and single-nucleus RNA-Seq toolbox for fresh and frozen human tumors. Nat Med. 2020 May;26(5):792–802.

**Second round of review**

**Reviewer 1**

The authors have satisfactorily addressed all of my concerns, and I appreciate their willingness to generate new data and analyses to improve their (already solid at the time of submission) manuscript. I fully support this manuscript's publication in Genome Biology -- and, assuming the editors and other reviewer agree, congrats!

**Reviewer 2**

The authors have reviewed the manuscript addressing my concerns. I am still of the opinion that a more thorough characterization of the cytometric, microscopic and molecular properties of DSP fixed cells would have provided valuable information but I realize that this is a lot of work and that this would delay the publication of this work. I appreciate the author’s efforts to comment on the applicability of Fix’n’cut. I have no further comments or questions.
